# Management of Canadian patients with refractory or relapsed diffuse large B-cell lymphoma in the real world: a subanalysis of the RE-MIND2 study

**DOI:** 10.1093/oncolo/oyaf330

**Published:** 2025-10-06

**Authors:** Anthea Peters, Grzegorz S Nowakowski, Rosy Dabas, Theresa Amoloja, Zhenyi Xue, Caroline Koch, Eva E Waltl, Isabelle Fleury

**Affiliations:** Department of Oncology, Cross Cancer Institute, University of Alberta, Edmonton, AB T6G 1Z2, Canada; Division of Hematology, Mayo Clinic, Rochester, MN 55905, United States; Medical Affairs - Hematology/Oncology, Incyte Biosciences Canada, Villle St Laurent, QC H4S 0A9, Canada; Global Epidemiology and RWE Statistics, Incyte Corporation, Wilmington, DE 19803, United States; Global Epidemiology and RWE Statistics, Incyte Corporation, Wilmington, DE 19803, United States; Medical Affairs - Hematology/Oncology, Incyte Biosciences Canada, Villle St Laurent, QC H4S 0A9, Canada; Global Medical Affairs, MorphoSys AG, Planegg 82152, Germany; Hematology-Oncology and Cell Therapy University Institute, Hôpital Maisonneuve-Rosemont, Montreal University, Montreal, QC H1T 2M4, Canada

**Keywords:** diffuse large B-cell lymphoma, Canada, real-world clinical trials, autologous transplantation, stem cell transplantation, CAR T-cell therapy

## Abstract

**Background:**

In the current Canadian treatment landscape for relapsed or refractory diffuse large B-cell lymphoma (R/R DLBCL), eligibility for autologous stem cell transplantation (ASCT) guides the choice of salvage treatment. CD19 chimeric antigen receptor T-cell (CAR-T) therapies have improved outcomes in patients with chemorefractory DLBCL, but access is limited to eligible patients. This subanalysis of the RE-MIND2 observational retrospective cohort study investigated treatment patterns for R/R DLBCL in Canada.

**Patients and methods:**

Data from patients enrolled in RE-MIND2 treated between 2010 and 2020 at 2 Canadian centers were retrospectively collected from health records. Descriptive statistics were used to analyze baseline characteristics, treatment initiated, and duration of treatment by line of therapy.

**Results:**

One hundred and nine patients were included; 74.2% of patients were eligible for ASCT as 2L therapy, and 45.4% received transplants. ASCT eligibility for third- (3L) and fourth-line (4L) therapy declined to 17.1% and 5.9%, respectively. Patients received a wide variety of treatments in 3 and 4L. CAR-T therapy became available in 3 and 4L by the end of 2019. Median durations of treatment were <2.6 months in all lines of therapy; median time to next treatment ranged from 3.4 months in 4L to 5.3 months in 2L.

**Conclusion:**

Results of our study support that ASCT-ineligible patients have a poor prognosis with conventional salvage chemotherapy. Before the availability of novel immunotherapies, no apparent standard of care was observed for Canadian patients with R/R DLBCL who were ineligible for or did not receive ASCT, especially after 2L treatment.

Implications for practice Our study identified a standard of care gap for patients with relapsed/refractory diffuse large B-cell lymphoma (R/R DLBCL) ineligible for autologous stem cell transplantation (ASCT). Canadian patients with R/R DLBCL were observed to have a short duration of treatment with conventional chemotherapy, supporting the importance of early and effective treatment due to the aggressive nature of the disease. Novel agents such as polatuzumab vedotin plus bendamustine and rituximab and tafasitamab plus lenalidomide, and positive results from pivotal trials with chimeric antigen receptor T-cell therapy and bispecific T-cell engagers, could change treatment practices for ASCT-ineligible patients with R/R DLBCL.

## Introduction

Diffuse large B-cell lymphoma (DLBCL) is the most common subtype of non-Hodgkin lymphoma (NHL), accounting for ∼30% of all cases.^1^ DLBCL represents a biologically heterogeneous group displaying a wide range of clinical presentations and outcomes.^1^ The combination of rituximab, cyclophosphamide, doxorubicin, vincristine, and prednisone (R-CHOP) is standard first-line (1L) therapy for DLBCL.^2-4^ However, 30%-40% of patients with DLBCL are either refractory to or relapse (R/R) following 1L treatment.^1^^,^^5^

For patients with R/R DLBCL, salvage therapy is guided by eligibility for high-dose chemotherapy and autologous stem cell transplantation (HDT-ASCT). In the current Canadian treatment landscape for R/R DLBCL, CD19 chimeric antigen receptor T-cell (CAR-T) therapies have recently become available for patients eligible for HDT-ASCT with primary refractory DLBCL or a relapse within 12 months of 1L treatment. For patients with later relapse, second-line (2L) treatment with platinum-based salvage chemotherapy followed by HDT-ASCT is recommended.^1-3^ However, at least half of patients with R/R DLBCL are ineligible for ASCT,^1^^,^^6^^,^^7^ and patients who do not receive HDT-ASCT have a median overall survival (OS) of ∼4-10 months with conventional salvage chemotherapy.^8-10^

CAR-T therapies are approved for patients with R/R DLBCL in 2L (axicabtagene ciloleucel in 2019 and lisocabtagene maraleucel in 2022)^11^^,^^12^ and third-line (3L) settings (tisagenlecleucel in 2018).^13^ However, access is restricted by the few numbers of CAR-T centers throughout Canada and the inherent limitations associated with CAR-T therapy, such as comorbidities, rapid progressive disease, and the need for traveling, among others.^14^ Polatuzumab vedotin plus bendamustine and rituximab (Pola-BR) was approved in 2020 in the 2L setting,^15^ and epcoritamab and glofitamab were approved in 2023, both in the 3L setting.^16^^,^^17^ Loncastuximab (CD19 targeted antibody-drug conjugate) was approved in 2025 for patients with R/R DLBCL in the 3L setting.^18^ Tafasitamab (CD19 monoclonal antibody) plus lenalidomide (tafa + len) was approved in the 2L setting for ASCT-ineligible patients with R/R DLBCL in 2021 based on the phase 2, single-arm registration trial, L-MIND (NCT02399085).^19^^,^^20^ Final results from the L-MIND study showed tafa + len had a manageable safety profile, and 58% of ASCT-ineligible patients with R/R DLBCL had an objective response. Most responders (72%) achieved a complete response and the median duration of response was not reached. Median progression-free survival and OS were 12 and 34 months, respectively.^21^

Real-world data on the management of patients with R/R DLBCL in Canada are very limited. RE-MIND2 (NCT04697160)^22^ was an observational retrospective cohort study designed to capture patient-level data for patients with R/R DLBCL to create a synthetic control arm to match the patient cohort that received tafa + len in L-MIND.^19^ Of 3454 total patients enrolled in RE-MIND2, 118 were Canadian, and 25 could be matched to the patient population enrolled in L-MIND.

Using data from Canadian patients with R/R DLBCL enrolled in the RE-MIND2 study, we conducted a descriptive *post hoc* subgroup analysis of the treatment landscape in this patient group between 2010 and 2020, a period before Pola-BR and tafa + len were available and/or reimbursed in Canada, and before CAR-T became more broadly available across the country. The objectives of this analysis were to describe the disease landscape, real-world patient characteristics, and management of patients with R/R DLBCL in Canada; to describe treatment patterns of patients from the time of initial diagnosis through different lines of therapy; and to identify treatment gaps to optimize patient care.

## Patients and methods

### Study design

The complete study design of the retrospective observational cohort study, RE-MIND2 (ClinicalTrials.gov, NCT04697160), has been reported previously.^22^ This analysis draws on data from 2 major Canadian academic centers (Cross Cancer Institute, Edmonton, AB, Canada, and Hôpital Maisonneuve-Rosemont, Montreal, QC, Canada) that participated in RE-MIND2. After assessment of eligibility criteria, data on patients diagnosed between 2010 and 2020 were retrospectively collected from health records. Data collected include demographic characteristics, date and histologic subtype of initial DLBCL diagnosis, history of cancers other than DLBCL, DLBCL therapies administered and their efficacy outcomes, treatment details (ie, start date, stop date, or discontinuation and reason), reasons for ASCT ineligibility, response assessment criteria used (Cheson 2014,^23^ 2007,^24^ or 1999 criteria^25^), Eastern Cooperative Oncology Group performance status (ECOG PS), bone marrow involvement, tumor biopsy information, and patient survival information (date and cause of death, and date of last patient contact). Patients were considered primary refractory if they had disease progression or a best response of less than partial response in 1L treatment, discontinued 1L treatment due to disease progression, or had disease progression or started 2L treatment ≤6 months after completing 1L treatment. An index date was assigned to each patient for each therapy line based on the first record of a systemically administered therapy for R/R DLBCL. For ASCT, the induction (salvage chemoimmunotherapy), stem cell collection, high dose preparative regimen (eg, carmustine, etoposide, cytarabine, and melphalan [BEAM]), and stem cell reinfusion, were considered a single line of therapy. This also applied to pre-planned consolidation of responding patients, where ASCT was regarded as a part of the systemic therapy line.

### Patient eligibility criteria

Eligibility criteria for RE-MIND2 were similar to those in L-MIND^19^; estimated propensity score–based 1:1 nearest neighbor matching was used to match patients in the RE-MIND2 observational cohort with patients in the L-MIND tafa + len cohort.^22^ Patients enrolled in RE-MIND2 were ≥18 years of age, had histologically confirmed DLBCL, and R/R disease, defined as receipt of ≥2 systemic therapies (including ≥1 anti−CD20 therapy); ≥1 line of therapy must have been recommended according to the National Comprehensive Cancer Network (2019) or European Society for Medical Oncology (2015) guidelines.^3^^,^^26^ Patients were required to have had a baseline tumor assessment, a valid index date for the given line of therapy, and 6 months of follow-up from the index date.

Exclusion criteria in RE-MIND2 were central nervous system lymphoma involvement at initial DLBCL diagnosis, treatment with CD19-targeted therapy or immunomodulatory drugs (eg, thalidomide or lenalidomide) as front-line DLBCL therapy, receipt of allogeneic stem cell transplantation, prior history of malignancies other than DLBCL in the preceding 5 years (excluding basal or squamous cell carcinoma of the skin; carcinoma in situ of the cervix, breast, or bladder; and prostate cancer of stage T1a or T1b), and prior treatment with tafa.

### Endpoints

The primary endpoint of RE-MIND2 was OS. Endpoints assessed in this analysis included duration of treatment and time to next treatment (TTNT). TTNT was defined as the time from the index date for a given line of therapy to the start of the next anti-DLBCL therapy or death due to any cause, whichever came first. Duration of study treatment exposure was defined as the duration from the index date for a given line of therapy to the end of study treatment for the same line of therapy.

### Statistical analysis

Descriptive statistics were used to analyze baseline characteristics, treatment initiated, and duration of treatment by line of therapy (2L, 3L, and/or 4L). In addition, patients were categorized as HDT-ASCT eligible or HDT-ASCT ineligible based on the opinion of the investigator as to whether the patient was a candidate for HDT-ASCT. Patients were also categorized into age groups (<65 vs. ≥65 years) to evaluate whether age impacted treatment choice and duration. OS and distribution of TTNT were estimated using the Kaplan−Meier method; medians were calculated along with 95% CIs using the method of Brookmeyer and Crowley.^27^

### Ethics approval

RE-MIND2 was conducted according to the International Conference on Harmonisation Good Pharmacoepidemiology Practice guidelines and the Declaration of Helsinki. The study protocol was approved by an independent ethics committee or institutional review board, and informed consent was obtained from all patients involved in the RE-MIND2 study, as previously reported.^22^

## Results

### Patient characteristics

**Figure 1.**
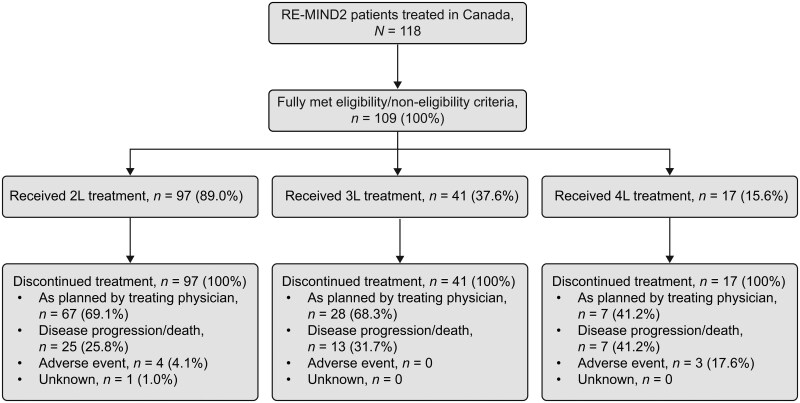
CONSORT diagram. Patients entered the study in the specified line of treatment and were not subsequently enrolled into later line(s) of therapy during the course of the study. 2L, second-line; 3L, third-line; 4L, fourth-line.

A total of 118 Canadian patients with R/R DLBCL were enrolled in the overall cohort of RE-MIND2; of these, 109 patients met eligibility criteria and were all included in this analysis. Patients were grouped by eligibility criteria based on prior line of therapy and included 97 (89.0%) who received 2L therapy, 41 (37.6%) who received 3L therapy, and 17 (15.6%) who received 4L therapy. Of note, patients could contribute to multiple lines of therapy. Patient disposition is shown in Figure 1. All patients had discontinued treatment at the end of the analysis window; most common reasons for treatment discontinuation were “as planned by the treating physician” (2L, 69.1%; 3L, 68.3%; 4L, 41.2%) and disease progression or death (2L, 25.8%; 3L, 31.7%; 4L, 41.2%). A total of 81 patients died during the study; the most common cause of death was disease progression (2L, 93.8%; 3L, 100.0%; 4L, 88.9%).

Patient demographics and disease characteristics by therapy line and ASCT eligibility status are shown in Table 1 and Supplementary Table S1. Median age at index date was 62.0 years (range, 26-86 years), and 69.7% of patients were male. A higher proportion of patients in later lines of therapy had ECOG PS ≥2 (2L, 11.3%; 3L, 14.6%; 4L, 23.5%). At initial diagnosis, 52 patients (47.7%) had germinal center B-cell (GCB) DLBCL, 31 (28.4%) had non-GCB DLBCL, and 26 (23.9%) had missing data; 20 patients (18.3%) had indolent NHL, which transformed to DLBCL. One patient had triple-hit lymphoma, and 6 patients had double-hit lymphoma, representing ∼6% of the cohort (Supplementary Table S2). Sixty-four patients (58.7%) had primary refractory DLBCL, including 33 patients (30.3%) with primary refractory disease while on 1L treatment, and 31 patients (28.4%) with early relapse ≤6 months following the end of 1L treatment. As expected, more patients receiving 3 and 4L therapy were refractory to the last prior therapy compared with those receiving 2L therapy (80.5% and 88.2% vs. 56.7%, respectively). This trend was also observed with refractoriness to rituximab/CD20 antibodies (82.9% and 82.4% vs. 56.7%, respectively).

**Table 1.** Patient demographics and baseline disease characteristics by line of therapy and ASCT eligibility status.

**Parameter (*N*** **=** **109)**	**2L (*n*** **=** **97)** ^a^			**3L (*n*** **=** **41)**			**4L (*n*** **=** **17)**		
	**ASCT eligible (*n*** **=** **72)**	**ASCT ineligible (*n*** **=** **24)**	Total	**ASCT eligible (*n*** **=** **7)**	**ASCT ineligible (*n*** **=** **34)**	Total	**ASCT eligible (*n*** **=** **1)**	**ASCT ineligible (*n*** **=** **16)**	Total
**Age at index date, years** ^**b**^									
Median (range)	58.5 (26-77)	71.5 (42-86)	63.0 (26-86)	58.0 (52-66)	64.0 (31-82)	63.0 (31-82)	58.0 (58-58)	62.0 (41-84)	62.0 (41-84)
≥65 years	21 (29.2)	18 (75.0)	40 (41.2)	1 (14.3)	16 (47.1)	17 (41.5)	0	5 (31.3)	5 (29.4)
**Sex, *n* (%)**									
Male	50 (69.4)	17 (70.8)	68 (70.1)	3 (42.9)	23 (67.6)	26 (63.4)	0	12 (75.0)	12 (70.6)
**IPI score, *n* (%)**									
0-2	18 (25.0)	1 (4.2)	19 (19.6)	2 (28.6)	5 (14.7)	7 (17.1)	0	3 (18.8)	3 (17.6)
3-5	11 (15.3)	8 (33.3)	19 (19.6)	1 (14.3)	7 (20.6)	8 (19.5)	0	7 (43.8)	7 (41.2)
Missing	43 (59.7)	15 (62.5)	59 (60.8)	4 (57.1)	22 (64.7)	26 (63.4)	1 (100.0)	6 (37.5)	7 (41.2)
**Number of extranodal sites, *n* (%)**									
0 or 1	29 (40.3)	11 (45.8)	41 (42.3)	5 (71.4)	16 (47.1)	21 (51.2)	0	8 (50.0)	8 (47.1)
≥2	17 (23.6)	5 (20.8)	22 (22.7)	0	7 (20.6)	7 (17.1)	0	4 (25.0)	4 (23.5)
Missing	26 (36.1)	8 (33.3)	34 (35.1)	2 (28.6)	11 (32.4)	13 (31.7)	1 (100.0)	4 (25.0)	5 (29.4)
**Ann Arbor disease stage, *n* (%)**									
I or II	9 (12.5)	2 (8.3)	11 (11.3)	0	6 (17.6)	6 (14.6)	0	2 (12.5)	2 (11.8)
III or IV	31 (43.1)	15 (62.5)	46 (47.4)	4 (57.1)	16 (47.1)	20 (48.8)	0	10 (62.5)	10 (58.8)
Missing	32 (44.4)	7 (29.2)	40 (41.2)	3 (42.9)	12 (35.3)	15 (36.6)	1 (100.0)	4 (25.0)	5 (29.4)
**ECOG PS, *n* (%)**									
0 or 1	39 (54.2)	11 (45.8)	51 (52.6)	2 (28.6)	13 (38.2)	15 (36.6)	0	8 (50.0)	8 (47.1)
≥2	10 (13.9)	1 (4.2)	11 (11.3)	1 (14.3)	5 (14.7)	6 (14.6)	0	4 (25.0)	4 (23.5)
Missing	23 (31.9)	12 (50.0)	35 (36.1)	4 (57.1)	16 (47.1)	20 (48.8)	1 (100.0)	4 (25.0)	5 (29.4)
**Prior CNS involvement, *n* (%)**									
Yes	3 (4.2)	3 (12.5)	6 (6.2)	0	5 (14.7)	5 (12.2)	0	1 (6.3)	1 (5.9)
No	69 (95.8)	21 (87.5)	91 (93.8)	7 (100.0)	29 (85.3)	36 (87.8)	1 (100.0)	15 (93.8)	16 (94.1)
**Primary refractoriness, *n* (%)**									
Yes	43 (59.7)	11 (45.8)	55 (56.7)	5 (71.4)	21 (61.8)	26 (63.4)	0	9 (56.3)	9 (52.9)
Primary progressive^c^	24 (33.3)	7 (29.2)	32 (33.0)	2 (28.6)	9 (26.5)	11 (26.8)	0	3 (18.8)	3 (17.6)
Early relapse^d^	19 (26.4)	4 (16.7)	23 (23.7)	3 (42.9)	12 (35.3)	15 (36.6)	0	6 (37.5)	6 (35.3)
No	29 (40.3)	13 (54.2)	42 (43.3)	2 (28.6)	13 (38.2)	15 (36.6)	1 (100.0)	7 (43.8)	8 (47.1)
**Refractoriness to last prior therapy, *n* (%)**									
Yes	43 (59.7)	11 (45.8)	55 (56.7)	6 (85.7)	27 (79.4)	33 (80.5)	1 (100.0)	14 (87.5)	15 (88.2)
No	29 (40.3)	13 (54.2)	42 (43.3)	1 (14.3)	7 (20.6)	8 (19.5)	0	2 (12.5)	2 (11.8)
**Rituximab and CD20 antibody refractoriness, *n* (%)**									
Yes	43 (59.7)	11 (45.8)	55 (56.7)	7 (100.0)	27 (79.4)	34 (82.9)	1 (100.0)	13 (81.3)	14 (82.4)
No	29 (40.3)	13 (54.2)	42 (43.3)	0	7 (20.6)	7 (17.1)	0	3 (18.8)	3 (17.6)

Abbreviations: 1L, first-line; 2L, second-line; 3L, third-line; 4L, fourth-line; ASCT, autologous stem cell transplantation; CNS, central nervous system; DLBCL, diffuse large B-cell lymphoma; ECOG PS, Eastern Cooperative Oncology Group performance status; IPI, international prognostic index; PD, progressive disease; PR, partial response; SD, stable disease.

a One patient who was ASCT ineligible received ASCT in 2L.

b Index date is the date recorded when treatment was initiated for the respective line (2L, 3L, or 4L).

c Primary progressive is defined as patients meeting ≥ 1 of the following criteria: (1) PD during the course of 1L treatment (systemic anti-DLBCL medication), that is, status at the end of 1L treatment is PD; (2) best response of less than PR (ie, PD or SD) in 1L treatment; or (3) reason for discontinuation of 1L treatment is “disease progression.”

d Early relapse is defined as patients receiving 2L treatment started within ≤6 months from the completion of 1L therapy.

**Figure 2.**
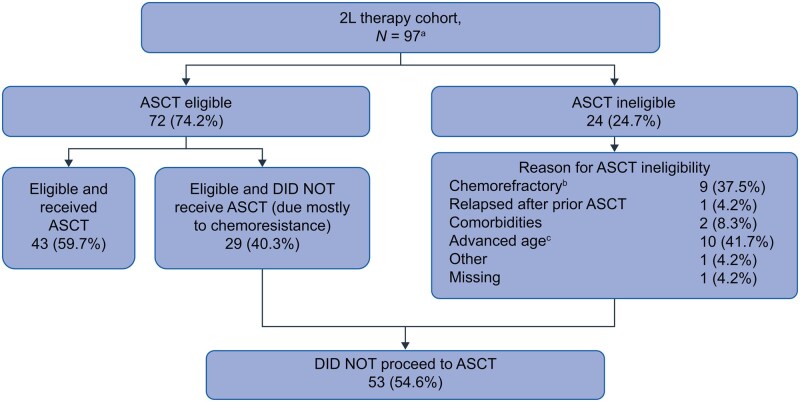
ASCT eligibility at the start of 2L therapy and reasons for ASCT ineligibility. ^a^One patient who was ASCT ineligible received ASCT in 2L. ^b^Defined as PD or SD as best response to chemotherapy. ^c^Median (range) age was 71.5 (42-86) years. 2L, second-line; ASCT, autologous stem cell transplantation; PD, progressive disease; SD, stable disease.

Most of the 2L patients were eligible for HDT-ASCT at the start of that treatment line (74.2%), whereas only 17.1% and 5.9% of patients were eligible for HDT-ASCT at the start of 3 and 4L treatment, respectively. The most common reasons patients were ineligible for HDT-ASCT were chemorefractoriness (38%, 53%, and 56%), advanced age (42%, 18%, and 13%), and relapse after prior ASCT (4%, 24%, and 31%) at the start of 2L, 3L, and 4L, respectively (Supplementary Table S3). Among the 97 patients in the 2L cohort, 54.6% did not proceed to ASCT (Figure 2).

### Treatment patterns (2010-2020)

Treatments administered to Canadian patients with R/R DLBCL between 2010 and 2020 were grouped into the following: HDT-ASCT; gemcitabine, and dexamethasone, and cisplatin (GDP) ± R; gemcitabine plus oxaliplatin with or without rituximab (GemOx ± R); ifosfamide, carboplatin, and etoposide (ICE) ± R; bendamustine; CAR-T; and others (including non-rituximab/rituximab-containing regimens, high-dose methotrexate, or other investigational therapies such as lenalidomide monotherapy).

HDT-ASCT was the intended treatment for 45.4% of patients in 2L. GDP ± R (irrespective of plan to proceed to ASCT) was the most prescribed 2L salvage chemotherapy (67/97 [69.1%]). Among the 60 patients considered HDT-ASCT eligible who received GDP ± R in 2L, 36 (60.0%) proceeded to ASCT in 2L, including 1 patient not eligible for HDT-ASCT who received HDT-ASCT and GDP + R. GDP ± R was received in 2L by 7/25 patients (28%) in the ASCT-ineligible cohort. In 3L, the largest group of patients (43.9%) received “other” treatments; 24.4% underwent CAR-T and 9.8% received ICE ± R (Table 2). In 4L, 41.2% of patients received “other” treatments, 29.4% received CAR-T, and 17.6% received bendamustine (Table 2). “Other” therapies were mostly chemotherapy, with a wide variety of regimens reported (Supplementary Table S4). All “other” treatments were reported in only 1 patient, apart from DHAP + R and etoposide, which were both reported in 2 patients (1 in 2L and 1 in 3L for DHAP + R; 2 in 3L for etoposide). Six patients had a non-chemotherapy “other” treatment, including idelalisib (1 patient in 2L), lenalidomide monotherapy (1 patient in 3L), and other investigational therapies as part of clinical trials (2 patients in 3L and 2 patients in 4L).

**Table 2.** Proportion of patients receiving each treatment and duration of treatment by line of therapy.

Treatment, *n* (%) (median [range] duration, days)	**2L (*n*** **=** **97)**	**3L (*n*** **=** **41)**	**4L (*n*** **=** **17)**
**HDT-ASCT** ^**a**^	44 (45.4)	3 (7.3)	1 (5.9)
	97 [48-335]	76 [48-78]	53 [53-53]
**GDP** **±** **R**	31 (32.0)	2 (4.9)	–
	38 [1-138]	61 [9-113]	–
**GemOx** **±** **R**	5 (5.2)	1 (2.4)	–
	22 [1-65]	15 [15-15]	–
**ICE** **±** **R**	2 (2.1)	4 (9.8)	1 (5.9)
	13 [1-25]	44 [1-92]	1 [1-1]
**Bendamustine**	5 (5.2)	3 (7.3)	3 (17.6)
	106 [43-148]	30 [1-145]	43 [35-78]
**CAR-T** ^b^	–	10 (24.4)	5 (29.4)
	–	–	–
**Others** ^c^	10 (9.3)	18 (43.9)	7 (41.2)
	15 [1-149]	47 [1-239]	43 [1-220]

Abbreviations: 2L, second-line; 3L, third-line; 4L, fourth-line; ASCT, autologous stem cell transplantation; CAR-T, chimeric antigen receptor T-cell; GDP ± R, cisplatin, gemcitabine, and dexamethasone with or without rituximab; GemOx ± R, gemcitabine plus oxaliplatin with or without rituximab; HDT, high-dose chemotherapy; ICE ± R, ifosfamide, carboplatin, and etoposide with or without rituximab.

a Duration of treatment for ASCT includes the duration of prior chemotherapy received.

b CAR-T was administered as a single infusion; therefore, no duration of treatment is given.

c Others included non-rituximab/rituximab-containing regimens, methotrexate, or other investigational therapies such as lenalidomide monotherapy.

**Figure 3.**
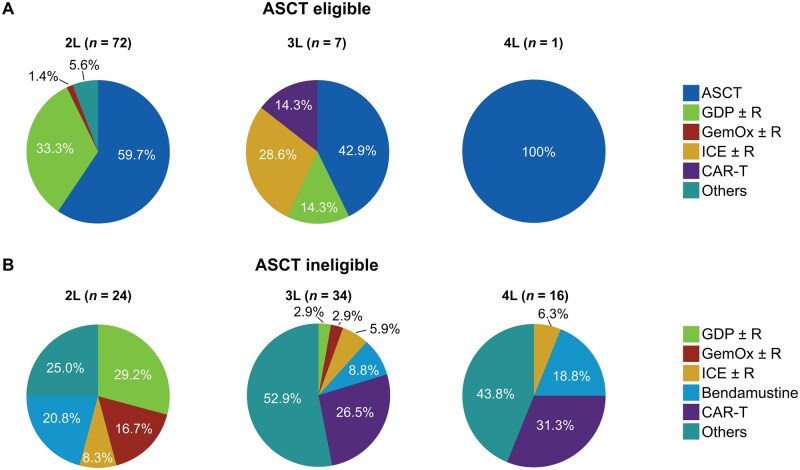
Treatment patterns among (A) HDT-ASCT–eligible and (B) HDT-ASCT–ineligible patients by line of therapy. GDP ± R includes patients who received GDP ± R as salvage therapy before ASCT but did not proceed to transplantation, and those who did not receive GDP ± R as salvage therapy. “Others” included non-rituximab/rituximab-containing regimens, or other investigational therapies such as lenalidomide monotherapy or Pola-BR; methotrexate was administered as part of another regimen but was also captured as a separate therapy for some patients and included in the “others” category. 2L, second-line; 3L, third-line; 4L, fourth-line; ASCT, autologous stem cell transplantation; CAR-T, chimeric antigen receptor T cell; GDP ± R, cisplatin, gemcitabine, and dexamethasone with or without rituximab; GemOx ± R, gemcitabine plus oxaliplatin with or without rituximab; HDT, high-dose chemotherapy; ICE ± R, ifosfamide, carboplatin, and etoposide with or without rituximab; pola-BR, polatuzumab vedotin plus bendamustine and rituximab.

Treatment patterns among HDT-ASCT–eligible and HDT-ASCT–ineligible patients showed a clear shift from standard of care (SOC) treatments after 2L, especially in HDT-ASCT–ineligible patients (Figure 3). In 2L, ∼40% (29/72) of HDT-ASCT–eligible patients did not proceed to transplant and received systemic therapies. In 3L, among HDT-ASCT–ineligible patients, “other” treatments were the most frequently used therapies (47.1%). CAR-T therapy was the second most common (26.5%) in 3L; overall, 15 patients received CAR-T therapy (10 in 3L and 5 in 4L), and 4 of them received CAR-T as part of a clinical trial.

**Figure 4.**
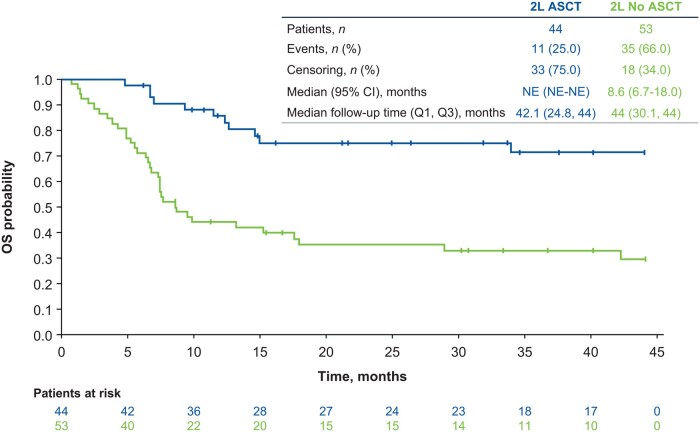
OS for patients who did or did not receive HDT-ASCT in 2L. 2L, second-line; ASCT, autologous stem cell transplantation; HDT, high-dose chemotherapy; NE, not estimable; OS, overall survival; Q1, first quartile; Q3, third quartile.

**Figure 5.**
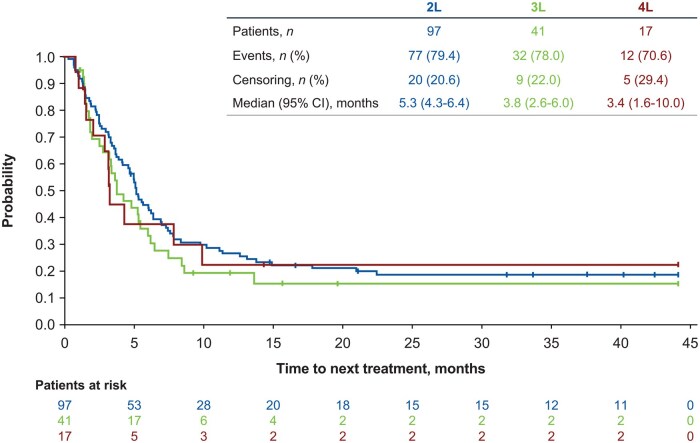
Time to next treatment among all patients (both eligible and ineligible for HDT-ASCT) by line of therapy. 2L, second-line; 3L, third-line; 4L, fourth-line; ASCT, autologous stem cell transplantation; HDT, high-dose chemotherapy.

The median duration of treatment exposure for patients not receiving HDT-ASCT ranged from 13 days (for ICE ± R) to 106 days (for bendamustine) in 2L, 15 days (for GemOx ± R) to 61 days (for GDP ± R) in 3L, and 1 day (for ICE ± R) to 43 days (for bendamustine and others) in 4L (Table 2). The median OS was not estimable for patients who received ASCT in 2L and 8.6 months for those who did not receive ASCT in 2L (Figure 4). Overall, age did not seem to affect treatment choice for 3L or 4L, with patients <65 and ≥65 years of age all receiving a wide range of treatments. The median TTNT among all patients (both eligible and ineligible for transplant) were 5.3, 3.8, and 3.4 months in 2L, 3L, and 4L, respectively (Figure 5).

All patients included in the analysis had tumor assessments. For most patients, tumor assessments were performed based on the Cheson 2014 criteria (78.4% in 2L, 65.9% in 3L, and 70.6% in 4L). One patient (5.9%) in 4L had assessments performed using the Cheson 2007 criteria. Tumor assessment criteria other than Cheson 2014, 2007, or 1999 were used in 18.6%, 29.3%, and 5.9% of 2, 3, and 4L patients, respectively. The method of tumor assessment was unknown in 3.1%, 4.9% and 17.6% of 2, 3, and 4L patients, respectively.

## Discussion

This descriptive analysis of the Canadian cohort of patients with R/R DLBCL in RE-MIND2 demonstrated that before the availability of novel immunotherapies, HDT-ASCT was the 2L SOC for eligible patients, while there was no established SOC for patients ineligible for HDT-ASCT.

Following 1L treatment, no consistent treatment approach was observed other than platinum-based salvage therapy and HDT-ASCT. Among patients receiving 2L treatment, GDP ± R was the most commonly used regimen (32%). Notably, 55% of patients in the 2L cohort did not proceed to ASCT, highlighting the need for alternate treatment strategies in this population. Patients who received ASCT in 2L showed improved OS compared with patients who did not receive ASCT in 2L. In 3 and 4L, CAR-T was used in 24% and 29% of patients, respectively, as it only became available in certain Canadian provinces starting in 2019. Given the study timeframe (2010-2020), treatment with CAR-T therapy in Canada was beginning and has resulted in improved outcomes for patients with R/R DLBCL after at least 2 prior lines of therapy. Reported complete response rates reach 78% with axicabtagene ciloleucel and 42% with tisagenlecleucel, with 12-month progression-free survival of 72% and 35%, respectively.^28^ “Other” treatments used in 3L and 4L relied mainly on clinical trials, highlighting an unmet need.

Previous studies have also demonstrated a variety of treatment approaches to R/R DLBCL in 2L and beyond. In a population-based study of 736 patients in Sweden, 35% of patients received an intensive chemotherapy regimen in 2L, 28% received a remission-inducing regimen, and 20% and 18% received palliative or no active treatment, respectively, with clear differences between those ≤70 and >70 years of age.^6^ In a US-based analysis of real-world outcomes in patients with R/R DLBCL who were ineligible for and/or failed ASCT, 41.3% received a bendamustine-based regimen, 37.1% received a gemcitabine-based regimen, and 21.7% received lenalidomide^29^; most patients also received rituximab. Nine different systemic 2L therapies were reported in a prospective observational study in the United States.^30^ In an analysis of 3L treatments in patients <65 years of age who failed 2L salvage regimens in the international CORAL study, 166/203 received 3L salvage treatment, including ICE-like (18.5%), DHAP-like (18%), and gemcitabine-containing regimens (13.8%); however, the greatest proportion of patients received “miscellaneous” therapy (31.9%).^9^ In a single-center, retrospective, observational, US-based study comparing 215 patients with R/R DLBCL treated with commercially available CAR-T or alternate therapies, 9 different 3L therapies were reported, as well as participation in a clinical trial.^31^ These studies suggest that physicians tailor available treatment options for R/R DLBCL for each patient, particularly after 2L, and consider age and frailty when choosing treatments.

A high percentage of patients (74.2%) in this Canadian cohort were eligible for HDT-ASCT; this is likely due to the relatively low median cohort age of 63 years, which is younger than the median age in the overall RE-MIND2 study (71.5 years), the RE-MIND study (71.5 years), and the L-MIND study (72 years).^19^^,^^22^^,^^32^ The age of the cohort of patients in the present study is also younger than in a Swedish population-based study (71 years), in which 66% of patients ≤70 years of age were unable to have HDT-ASCT within 12 months of relapse.^6^ However, the median age of patients in this analysis is equal to that of a population-based study in Japan (63 years), in which 75% of patients were unable to receive HDT-ASCT.^33^ In the present analysis, ∼40% of patients who were identified as HDT-ASCT eligible did not proceed to receive HDT-ASCT. It has also been observed in other studies that a substantial proportion of patients with R/R DLBCL who were considered eligible for ASCT did not proceed to transplant, ranging from 52% to 84%^6^^,^^7^^,^^30^^,^^33-36^; the most common reason for being unable to proceed to transplant is due to inadequate response to salvage therapy.^7^^,^^30^^,^^36^ The relatively young cohort age in the RE-MIND2 Canadian subgroup analysis and the lack of alternative therapies are possible contributors to the slightly lower proportion (40%) of patients unable to proceed to transplant.

In this study, OS and median duration of treatment exposure in patients not receiving HDT-ASCT were short (8.6 months and ≤3.5 months, respectively) and median TTNT was 5.3 months for 2L, 3.8 months for 3L, and 3.4 months for 4L. The limited median OS for patients who could not receive HDT-ASCT in this study is of a similar magnitude to the OS reported in other studies, which ranges from 4.4 to 10.4 months (depending on study and treatment).^6^^,^^9^^,^^10^^,^^29-31^^,^^37^

Our analysis has inherent limitations. The Canadian cohort included patient data from only 2 Canadian hospitals and a relatively small sample size of 109 patients; therefore, this study may not reflect the management of patients with DLBCL throughout Canada and may be prone to selection and reporting bias. Furthermore, data were collected only at a single time point, at the start of each line of therapy; therefore, longitudinal data for individual patients are not available throughout the analysis. This is compounded by some data being missing, both from the overall RE-MIND2 analysis and this Canadian cohort. Additionally, patients in this study were diagnosed between 2010 and 2020, during which time CAR-T was available at only 1 of the centers in this study; since then, CAR-T and other new treatments have become more widely available in Canada. A further limitation is that although most response assessments were based on the Cheson 2014 criteria, for patients assessed before 2014, responses were not retrospectively updated. Further retrospective Canadian registry analyses of patients with DLBCL are needed to confirm these findings.

## Conclusion

Understanding country-specific patterns of care and outcomes is critical in identifying unmet needs. At the time of analysis, salvage therapy relied on conventional chemotherapy or clinical trials if available for eligible patients. Based on the wide range of treatment approaches prescribed to Canadian patients enrolled in this cohort, there is a clear gap in SOC for patients with R/R DLBCL ineligible for HDT-ASCT, with no availability of CAR-T in 2L. The observation of the short duration of treatment in these patients with R/R DLBCL supports the importance of treating patients early with more effective agents, due to the severity and aggressive nature of the disease. The treatment landscape in patients with R/R DLBCL has changed since the time of analysis, with increased availability of novel agents in 2L for patients with R/R DLBCL ineligible for HDT-ASCT, such as Pola-BR since 2020 (available everywhere in Canada except Quebec) and tafa + len since 2021 (reimbursed since August 2023 in Quebec), and promising results from pivotal trials with CAR-T and bispecific T-cell engagers. The challenges of treatment sequencing, patient selection, and clinical decision-making in HDT-ASCT–ineligible patients are still relevant today despite evolving therapies. Additionally, there is limited access to CAR-T therapies and bispecific T-cell engagers in rural areas of Canada. Insights from this analysis can inform ongoing clinical strategies by demonstrating the impact of different treatment pathways on patient outcomes.

## Supplementary Material

oyaf330_Supplementary_Data

## Data Availability

Incyte Corporation (Wilmington, DE, USA) is committed to data sharing that advances science and medicine while protecting patient privacy. Qualified external scientific researchers may request anonymized datasets owned by Incyte for the purpose of conducting legitimate scientific research. Researchers may request anonymized datasets from any interventional study (except phase I studies) for which the product and indication have been approved on or after January 1, 2020, in at least 1 major market (eg, US, EU, and JPN). Data will be available for request after the primary publication or 2 years after the study has ended. Information on Incyte’s clinical trial data sharing policy and instructions for submitting clinical trial data requests are available at: https://www.incyte.com/Portals/0/Assets/Compliance%20and%20Transparency/clinical-trial-data-sharing.pdf? ver=2020-05-21-132838-960.
