# Supplementary material for: Management of Canadian patients with refractory or relapsed diffuse large B-cell lymphoma in the real world: a subanalysis of the RE-MIND2 study
Source: Oncologist. 2025 Oct 6;30(11):oyaf330. doi: 10.1093/oncolo/oyaf330 (PMC12605763; doi:10.1093/oncolo/oyaf330)
Supplement: oyaf330_Supplementary_Data [file oyaf330_supplementary_data.zip › Peters_RE-MIND2 Ms_The Oncologist Resub_Suppl_27Jun2025.pdf]

Supplementary Material

Management of Canadian Patients With Refractory or Relapsed Diffuse Large B-Cell Lymphoma in the Real World: A Subanalysis of the RE-MIND2 Study

Anthea Peters[1]*, Grzegorz S. Nowakowski[2], Rosy Dabas[3], Theresa Amoloja[4], Zhenyi Xue[4], Caroline Koch[3], Eva E. Waltl[5], Isabelle Fleury[6]

[1]Department of Oncology, Cross Cancer Institute, University of Alberta, 11560 University Avenue, Edmonton, AB, T6G 1Z2 Canada

[2]Division of Hematology, Mayo Clinic, 200 First Street SW, Rochester, MN 55905, USA

[3]Incyte Biosciences Canada, 6500 Trans-Canada Highway, Suite 400, Pointe-Claire, QC H9R 0A5, Canada

[4]Incyte Corporation, 1801 Augustine Cut-off, Wilmington, DE 19803, USA

[5]MorphoSys AG, Semmelweisstr. 7, 82152 Planegg, Germany

[6]Hôpital Maisonneuve-Rosemont, Montreal University, 5415 Assomption Boulevard, Montreal, QC H1T 2M4, Canada

Supplementary Table S1. Baseline disease characteristics by line of therapy and ASCT eligibility status

	**2L (*n* = 97)[a]**			**3L (*n* = 41)**			**4L (*n* = 17)**		
**Parameter (*N* = 109)**	**ASCT eligible (*n* = 72)**	**ASCT ineligible (*n* = 24)**	**Total**	**ASCT eligible (*n* = 7)**	**ASCT ineligible (*n* = 34)**	**Total**	**ASCT eligible (*n* = 1)**	**ASCT ineligible (*n* = 16)**	**Total**
Neutropenia (<1.5 × 10^9/L)									
Yes	4 (5.6)	0	4 (4.1)	0	1 (2.9)	1 (2.4)	0	0	0
No	56 (77.8)	21 (87.5)	78 (80.4)	7 (100.0)	29 (85.3)	36 (87.8)	1 (100.0)	11 (68.8)	12 (70.6)
Missing	12 (16.7)	3 (12.5)	15 (15.5)	0	4 (11.8)	4 (9.8)	0	5 (31.3)	5 (29.4)
Anemia (hemoglobin <10 g/dL)									
Yes	8 (11.1)	4 (16.7)	12 (12.4)	2 (28.6)	4 (11.8)	6 (14.6)	0	0	0
No	52 (72.2)	17 (70.8)	70 (72.2)	5 (71.4)	26 (76.5)	31 (75.6)	1 (100.0)	11 (68.8)	12 (70.6)
Missing	12 (16.7)	3 (12.5)	15 (15.5)	0	4 (11.8)	4 (9.8)	0	5 (31.3)	5 (29.4)
Elevated LDH (>ULN), *n* (%)									
Yes	32 (44.4)	12 (50.0)	45 (46.4)	6 (85.7)	18 (52.9)	24 (58.5)	1 (100.0)	8 (50.0)	9 (52.9)
No	27 (37.5)	9 (37.5)	36 (37.1)	1 (14.3)	9 (26.5)	10 (24.4)	0	2 (12.5)	2 (11.8)
Missing	13 (18.1)	3 (12.5)	16 (16.5)	0	7 (20.6)	7 (17.1)	0	6 (37.5)	6 (35.3)

Abbreviations: 2L, second-line; 3L, third-line; 4L, fourth-line; ASCT, autologous stem cell transplantation; LDH, lactate dehydrogenase; ULN, upper limit of normal.

[a]One patient who was ASCT ineligible received ASCT in 2L.

Supplementary Table S2. Genetic rearrangements in all patients.

**Rearrangements, *n* (%)**	**All systemic therapies pooled (*N* = 109)**
Triple-hit lymphoma (MYC, BCL-2, and BCL-6 rearrangements)	1 (0.9)
Double-hit lymphoma (MYC with BCL-2 or BCL-6 rearrangements)	6 (5.5)
MYC and BCL-2 rearrangements	4 (3.7)
MYC and BCL-6 rearrangements	2 (1.8)
MYC rearrangements only	3 (2.8)
BCL-2 rearrangements only	4 (3.7)
BCL-6 rearrangements only	1 (0.9)
No rearrangements	92 (84.4)
Missing	2 (1.8)

Supplementary Table S3. Summary of HDT-ASCT eligibility at the start of the given line of therapy and reason for HDT-ASCT ineligibility.

**Parameter (*N* = 109)**	**2L (*n* = 97)**	**3L (*n* = 41)**	**4L (*n* = 17)**
Patients eligible for HDT-ASCT, *n* (%)[a]			
Yes	72 (74.2)	7 (17.1)	1 (5.9)
No	24 (24.7)	34 (82.9)	16 (94.1)
Unknown	1 (1.0)	0	0
Reason for HDT-ASCT ineligibility, *n* (%)			
Chemorefractory[b]	9 (37.5)	18 (52.9)	9 (56.3)
Relapsed after prior ASCT	1 (4.2)	8 (23.5)	5 (31.3)
Comorbidities	2 (8.3)	1 (2.9)	0
Advanced age	10 (41.7)	6 (17.6)	2 (12.5)
Patient refusal	0	0	0
Other	1 (4.2)	0	0
Missing	1 (4.2)	1 (2.9)	0

Abbreviations: 2L, second-line; 3L, third-line; 4L, fourth-line; ASCT, autologous stem cell transplantation; HDT, high-dose chemotherapy; PD, progressive disease; SD, stable disease.

[a]One patient who was ASCT ineligible received ASCT in 2L.

[b]Defined as PD or SD as best response to chemotherapy.

Supplementary Table S4. Other therapies[a] received by line of therapy.

Therapy, *n* (%)	2L (*n* = 97)	3L (*n* = 41)	4L (*n* = 17)
Received another therapy	10 (10.3)	18 (43.9)	7 (41.2)
Investigational	–	2 (4.9)	2 (11.8)
DHAP-R	1 (1.0)	1 (2.4)	–
Etoposide	–	2 (4.9)	–
Ara-C-dexamethasone-oxaliplatin	–	–	1 (5.9)
Ara-C-dexamethasone-oxaliplatin-R	–	–	1 (5.9)
Ara-C-methotrexate	–	1 (2.4)	–
Carboplatin-dexamethasone-gemcitabine-R	1 (1.0)	–	–
CEPP	–	1 (2.4)	–
CHOP-CVP-R-venetoclax	–	1 (2.4)	–
Cisplatin-cyclophosphamide-dexamethasone-etoposide-R	–	1 (2.4)	–
Cisplatin-cyclophosphamide-etoposide-R	1 (1.0)	–	–
Cyclophosphamide-etoposide-prednisone-procarbazine	–	1 (2.4)	–
Cytarabine-dexamethasone-HD-Ara-cytarabine-oxaliplatin	1 (1.0)	–	–
DA-EPOCH	1 (1.0)	–	–
Dexamethasone-etoposide	–	–	1 (5.9)
Dexamethasone-gemcitabine	1 (1.0)	–	–
Gemcitabine	–	1 (2.4)	–
HD cytarabine	–	1 (2.4)	–
Ibrutinib-methotrexate	–	–	1 (5.9)
Idelalisib	1 (1.0)		
Lenalidomide monotherapy	–	1 (2.4)	–
Methotrexate HD	1 (1.0)	1 (2.4)	–
Methotrexate-rituximab	–	–	1 (5.9)
R-CEOP	–	1 (2.4)	–
R-CHOP	1 (1.0)	–	–
R-DHAP-etoposide-ifosfamide-methotrexate	1 (1.0)	–	–
R-hyper CVAD	–	1 (2.4)	–

R-IVAC	–	1 (2.4)	–
Rituximab	–	1 (2.4)	–

Abbreviations: 2L, second-line; 3L, third-line; 4L, fourth-line; Ara-C, cytarabine; CEOP, cyclophosphamide, etoposide, vincristine, and prednisone; ASCT, autologous stem cell transplantation; CEPP, cyclophosphamide, etoposide, procarbazine, and prednisone; CHOP, cyclophosphamide, doxorubicin, vincristine, and prednisone; hyper CVAD, hyperfractionated cyclophosphamide, vincristine, doxorubicin, and dexamethasone; CVP, cyclophosphamide, vincristine, and prednisone; DA-EPOCH, etoposide, prednisone, vincristine, cyclophosphamide, and doxorubicin; DHAP, dexamethasone, high-dose cytarabine, and cisplatin; HD, high dose; IVAC, ifosfamide, etoposide, and cytarabine; R, rituximab.

[a]Patients who received ASCT with another therapy are not shown.
